# Leaf Epidermis: The Ambiguous Symplastic Domain

**DOI:** 10.3389/fpls.2021.695415

**Published:** 2021-07-29

**Authors:** Olga V. Voitsekhovskaja, Anna N. Melnikova, Kirill N. Demchenko, Alexandra N. Ivanova, Valeria A. Dmitrieva, Anastasiia I. Maksimova, Gertrud Lohaus, A. Deri Tomos, Elena V. Tyutereva, Olga A. Koroleva

**Affiliations:** ^1^Komarov Botanical Institute, Russian Academy of Sciences, Saint Petersburg, Russia; ^2^Department of Plant Biochemistry, Albrecht von Haller Institute for Plant Sciences, Göttingen, Germany; ^3^Saint Petersburg State University, Saint Petersburg, Russia; ^4^Molecular Plant Research/Plant Biochemistry, School of Mathematics and Natural Sciences, University of Wuppertal, Wuppertal, Germany; ^5^School of Biological Sciences, Bangor University, Bangor, United Kingdom

**Keywords:** *Alonsoa meridionalis*, *Asarina barclaiana*, *Hordeum vulgare*, *Solanum tuberosum*, leaf epidermis, phloem loading mode, secondary plasmodesmata, single cell sampling

## Abstract

The ability to develop secondary (post-cytokinetic) plasmodesmata (PD) is an important evolutionary advantage that helps in creating symplastic domains within the plant body. Developmental regulation of secondary PD formation is not completely understood. In flowering plants, secondary PD occur exclusively between cells from different lineages, e.g., at the L1/L2 interface within shoot apices, or between leaf epidermis (L1-derivative), and mesophyll (L2-derivative). However, the highest numbers of secondary PD occur in the minor veins of leaf between bundle sheath cells and phloem companion cells in a group of plant species designated “symplastic” phloem loaders, as opposed to “apoplastic” loaders. This poses a question of whether secondary PD formation is upregulated in general in symplastic loaders. Distribution of PD in leaves and in shoot apices of two symplastic phloem loaders, *Alonsoa meridionalis* and *Asarina barclaiana*, was compared with that in two apoplastic loaders, *Solanum tuberosum* (potato) and *Hordeum vulgare* (barley), using immunolabeling of the PD-specific proteins and transmission electron microscopy (TEM), respectively. Single-cell sampling was performed to correlate sugar allocation between leaf epidermis and mesophyll to PD abundance. Although the distribution of PD in the leaf lamina (except within the vascular tissues) and in the meristem layers was similar in all species examined, far fewer PD were found at the epidermis/epidermis and mesophyll/epidermis boundaries in apoplastic loaders compared to symplastic loaders. In the latter, the leaf epidermis accumulated sugar, suggesting sugar import from the mesophyll *via* PD. Thus, leaf epidermis and mesophyll might represent a single symplastic domain in *Alonsoa meridionalis* and *Asarina barclaiana*.

## Introduction

In land plants, cells can be connected by primary and/or secondary plasmodesmata (PD). Primary PD develop during cytokinesis and thus connect “sister cells” of the same cell lineage. In contrast, secondary PD develop *de novo*, i.e., post-cytokinetically, and can occur between “sister cells” as well as between cells which belong to different cell lineages. The ability to specifically enhance the extent of symplastic connectivity at any cell border by means of secondary PD formation represents an important evolutionary advantage, and not all vascular plants are able to develop secondary PD (Evkaikina et al., 2014). The exact mechanisms of the formation of secondary PD and the regulation of this process in different taxa of land plant are far from being completely understood.

In flowering plants, one of the best characterized examples of enhanced secondary PD formation is found in the minor veins of leaf species designated as symplastic phloem loaders (Gamalei, 1991; Van Bel and Gamalei, 1992). In symplastic phloem loaders, transfer of assimilates from the mesophyll into the phloem is assisted by highly developed PD at the boundary between bundle sheath cells and phloem companion cells. As these PD appear during the maturation of the leaf, i.e., after cell divisions have been completed, they represent secondary PD. The number of these PD can be very high in symplastic phloem loaders, especially in species that contain phloem companion cells of the “intermediary cell” and in “intermediary-cell-like” types (Gamalei, 1991; Batashev et al., 2013).

Several studies have suggested that apoplastic phloem loaders represent species with generally low PD frequencies between leaf cells and tissues, while species with generally abundant PD tend to represent symplastic phloem loaders (Gamalei, 1995; Turgeon and Medville, 2004). The overall symplastic connectivity of the cells of the leaf lamina has been proposed to correlate with the number of PD connecting bundle sheath and companion cells in the minor veins of leaf (Gamalei, 1995). This hypothesis was corroborated by the finding that the frequencies of PD between mesophyll cells correlate with the plasmodesmal frequencies at the phloem/mesophyll interface (considered as the indicator of the phloem loading mode, symplastic versus apoplastic) in several species as shown by Turgeon and Medville (2004). However, the question arises whether the high abundance of secondary PD across the mesophyll/phloem boundary of symplastic phloem loaders represents a systemic phenomenon of increased formation of secondary PD. At present, no technique is available to distinguish between secondary and primary PD at the cytological level. Thus, only the PD found between cells derived from different cell lineages, i.e., cells that never had a common division wall, can be safely considered as secondary, while boundaries between the cells of the same lineage are interpreted to contain a mixed population of both primary and secondary PD (Fitzgibbon et al., 2013).

Here, we compared the PD distribution between cells and cell layers in the leaves of four species from the following four different families: barley (*Hordeum vulgare*, Poaceae), potato (*Solanum tuberosum*, Solanaceae), *Alonsoa meridionalis* (Scrophulariaceae), and *Asarina barclaiana* (Plantaginaceae, formerly Scrophulariaceae). Barley and potato represent apoplastic phloem loaders (Riesmeier et al., 1994; Botha and Cross, 1997), while in *A. meridionalis* and *A. barclaiana*, phloem loading has a strong symplastic component (Voitsekhovskaja et al., 2006, 2009). We considered PD between leaf epidermis and mesophyll as secondary because epidermis and mesophyll originate from different cell lineages corresponding to the L1 and L2 layers, respectively in the leaf primordium (Satina and Blakeslee, 1941; Kang and Dengler, 2018). Moreover, in shoot apices of both *A. meridionalis* and *A. barclaiana*, the one-layered tunica of the meristem continuous with the protoderm of leaf primordia can be clearly distinguished, suggesting that like in many other species, cells of this layer represent an independent cell lineage also in *A. meridionalis* and *A. barclaiana* (Supplementary Figure 1). The PD between mesophyll cells as well as the PD between epidermal cells were considered as a mixture of primary and secondary PD, although some studies indicated that PD between mesophyll cells are predominantly primary (Oparka et al., 1999). To investigate the distribution of symplastic connections in leaf lamina, we labeled PD with antibodies raised against the PD-specific proteins, such as myosin VIII and calreticulin, respectively (Radford and White, 1998; Baluska et al., 1999; Reichelt et al., 1999; Schubert et al., 2013; Demchenko et al., 2014). We also compared the distribution of PD between cells and cell layers, L1, L2, and L3 in the shoot apical meristems (SAMs) of the same species using TEM. The results showed that the symplastic connectivity between cells of the epidermis, as well as at mesophyll/epidermis boundaries, is higher in the leaves of the symplastic loaders, *A. meridionalis* and *A. barclaiana*, compared to the apoplastic loaders, barley and potato, while no significant differences were detected for other cell boundaries. Moreover, in the species studied, the level of symplastic connectivity between mesophyll and epidermis was correlated with the ability of the latter to serve as a transient storage compartment for soluble sugars. Thus, in *A. meridionalis* and *A. barclaiana*, the leaf epidermis seems to form a symplastic domain together with the mesophyll.

## Materials and Methods

### Plant Material

*Alonsoa meridionalis* O. Kuntze (Scrophulariaceae) and *Asarina barclaiana* Pennell (Plantaginaceae) were grown on pot soil in a controlled growth chamber (Sanyo Gallenkamp, Loughborough, Leicester UK) at 20°C under a 16 h light/8 h dark cycle and a photon flux of 500 μmol m^−2^ s^−1^ and 0.035% CO_2_. Barley (*Hordeum vulgare* L.) was grown hydroponically at similar conditions. Potato plants (*Solanum tuberosum* L. cv. Désirée) were grown in a greenhouse under 12 h of supplemented artificial light of 400 μmol m^−2^ s^−1^ at 26°C and 12 h of dark at 18°C. All studies on whole leaves and on single cell samples were performed using mature fully expanded leaves (usually the third leaf from the top of the shoot). Samples of leaf tissue for immunolocalization studies were taken from the middle parts of the leaves. For TEM analyses of shoot apices, all species were sown from seeds and grown on soil in greenhouse under artificial light of 150 μmol m^−2^ s^−1^, at 16 h light/8 h dark cycle, at 21°C in the light and at 19°C in the dark. Apices of 14–30 day-old seedlings were fixed.

### Inhibition of Assimilate Export From the Leaves

Leaves of *A. meridionalis* and *A. barclaiana* were detached from the plants 3 h after the beginning of the light period and placed in continuous light conditions with a photon flux of 150 μmol m^−2^ s^−1^ for 24 h, while the petioles were kept in a 2 mM CaCl_2_ solution to favor the sealing of the phloem with callose (King and Zeevaart, 1974). Sugar concentrations were determined in single cell samples from leaves and in extracts of whole leaves after 24 h of light exposure.

### Sampling of Single Cells

Single cell sap was extracted from individual cells from the upper epidermis and palisade mesophyll by glass microcapillary technique (Tomos et al., 1994). Prior to use, a microcapillary was back-filled with low-viscosity water-saturated paraffin oil (Sigma). Ejection of the single cell sample under oil allowed the determination of sugars (glucose, fructose, and sucrose) by an enzymatic assay described by Koroleva et al. (1997, 1998). The measurements were highly reliable in a concentration range between 2 and 200 mM.

### Extraction and Analysis of Carbohydrates

Soluble carbohydrates were extracted from leaves with 80% ethanol at 80°C for 1 h. The extraction was repeated two times, and the extracts were combined, vacuum-dried, dissolved in ultra-pure water (Millipore), syringe-filtrated (0.45 μm cellulose-acetate; Schleicher and Schuell, Dassel, Germany), and stored at −80°C. For carbohydrate analysis by high-performance liquid chromatography (HPLC), an anion exchange column (CarboPAC10; Dionex Corp, Sunnyvale, CA, USA) was used. The column was eluted isocratically with 80 mM NaOH (J.T. Baker, England) with a flow rate of 1 ml min^−1^ using the LC-9A pump from Shimadzu (Kyoto, Japan). Sugars were detected by a thin layer of amperometric cell (ESA, Model 5200, Bedford, United States) with a gold electrode and a pulse amperometric detector (Coulochem II, Bedford, USA). The evaluation of chromatograms was performed using the integration program Peaknet 5.1 (Dionex, Idstein, Germany).

### Transmission Electron Microscopy of Shoot Apices

Shoot apices were fixed with 2.5% glutaraldehyde in 0.1 M potassium phosphate buffer pH 7.2 and post-fixed in 1% osmium tetroxide in the same buffer. During dehydration in a graded ethanol series followed by a graded acetone series, the material was stained *en bloc* with 1% uranyl acetate in 70% ethanol for 1 h, and then embedded in epon resin (Sigma Aldrich, MO, United States). Ultrathin sections (60–70 nm) were cut with a diamond knife (Diatome, Switzerland) using a Leica EM UC7 ultramicrotome (Leica Microsystems, Wetzlar, Germany), double stained on grids with 1% uranyl acetate and 3% lead citrate, and observed at 120 kV with a Libra 120 Plus electron microscope (Zeiss, Germany). The PD connecting cells of L1, L2, or L3 layers were counted manually in three apices for each species. For each apex, 8–25 cells per cell boundary (L1/L1, L1/L2, L2/L2, L2/L3, and L3/L3, respectively) were analyzed.

### Immunolocalization of Myosin VIII and Calreticulin in PD

**Figure 1:**
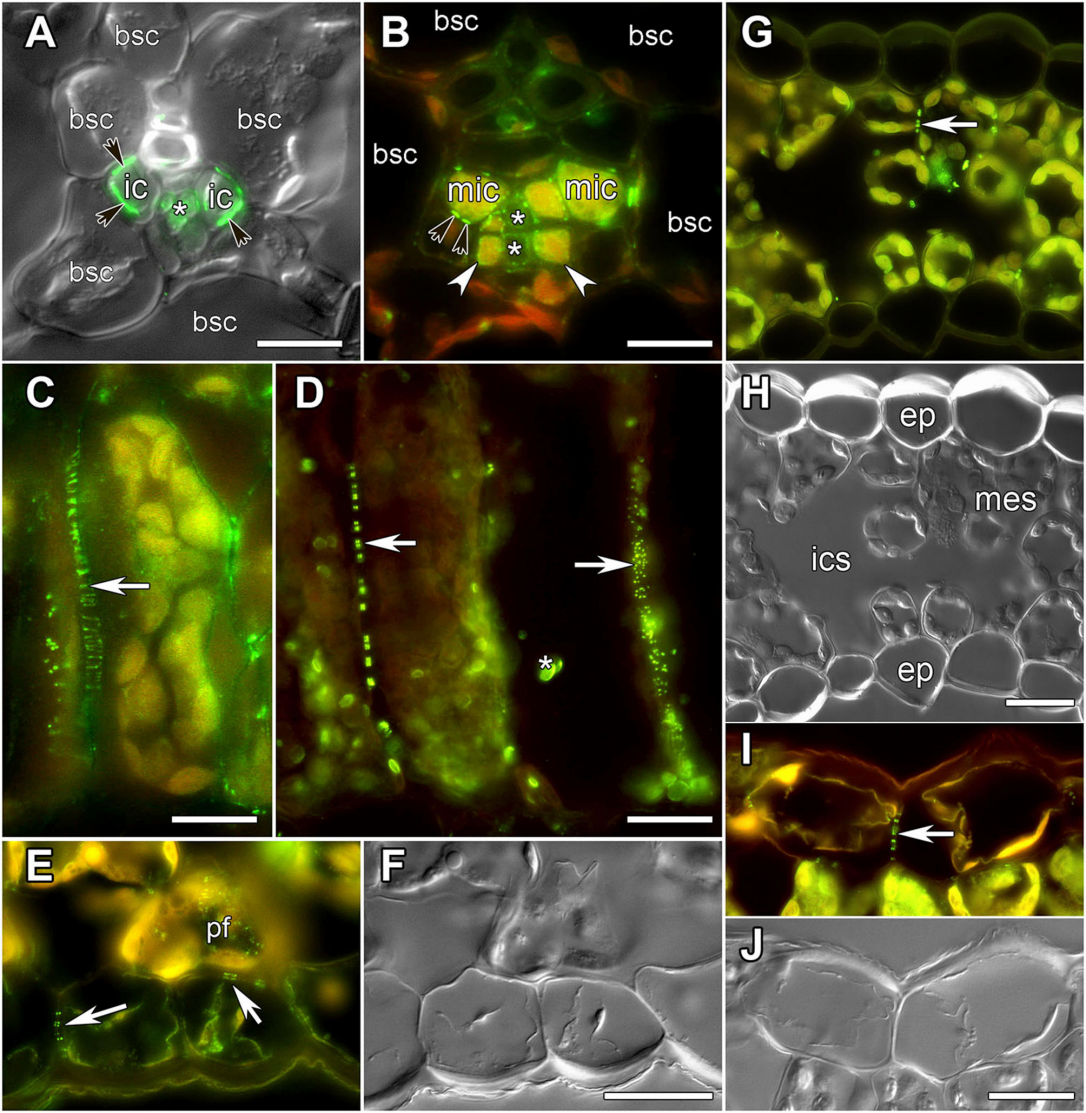
Images of leaf sections obtained using either differential interference contrast **(A,F,H,J)** or epifluorescent microscopy **(A**–**E,G,I)** showing labeling of PD by immunolocalization of calreticulin **(A–D)** or myosin VIII **(E–J)**, respectively. **(A,C,I,J)** *Alonsoa meridionalis*; **(B,E,F)** *Asarina barclaiana*; **(D)** potato; **(G,H)** barley. **(A,B)** Minor veins. bsc, bundle sheath cell; ic, intermediary companion cells in *A. meridionalis*; mic, modified intermediary cells in *A. barclaiana*; white arrows point to transfer cells in *A. barclaiana*; black arrows point to PD fields between intermediary cells and bundle sheath cells in *A. meridionalis* and between modified intermediary cells and bundle sheath cells in *A. barclaiana*, respectively; asterisks mark sieve elements. **(C,D)** palisade mesophyll cells in *A. meridionalis* **(C)** and potato **(D)**. Arrow points to PD; asterisk marks non-specific chloroplast labeling in potato **(D)**. **(E,F)** Part of an *A. barclaiana* leaf showing the lower epidermis. Arrows point to PD; pf, pitfield. **(G,H)** Transverse section through a barley leaf. ep, epidermal cell; mes, mesophyll cell; ics, intercellular space; arrows point to PD. **(I,J)** Part of an *A. meridionalis* leaf showing the upper epidermis. Arrows point to PD. Size bars: 10 μm **(A,B)**, 20 μm **(C–J)**.

Fixation, embedding, and sectioning of mature leaves were performed as described by Stumpe et al. (2006). The anti-myosin VIII-antibody (Reichelt et al., 1999) was diluted in the ratio, 1:100 in tris-buffered saline (TBS) containing 1% (w/v) bovine serum albumin (BSA). The anti-calreticulin antibody (Baluska et al., 1999) was diluted in the ratio, 1:200 in the same buffer. In negative controls, the primary antibody was omitted. The secondary antibody, goat anti-rabbit Alexa Fluor 488 (Molecular Probes, Eugene, OR, USA), was diluted in the ratio, 1:500 in TBS with 1% BSA. Semi-thin sections (8–10 μm) were mounted using the ProLong Gold Antifade kit (Molecular Probes, OR, United States). Immunofluorescence was detected using a BX51 microscope (Olympus Deutschland GmbH, Hamburg, Germany). Images were captured with an objective UPlanApo 40×/0.85 ∞/0.11–0.23 using a ColorView II digital camera and Cell F^*^ image analytical software (Olympus Soft Imaging Solutions, Münster, Germany). Images shown in Figures 1A,B were obtained using AxioImager.Z1 Microscope (Carl Zeiss, Goettingen, Germany) and UPlanFI 100×/ 1.30 Oil ∞/0.17 C1 objective, an Axiocam 506 color digital camera and ZEN microscope software v. 3.3. Supplementary Videos 1–8 showing views of different cell junctions in leaves of the species under study were recorded using the same microscope.

### Transmission Electron Microscopy of Semi-Thin Sections Used for Immunohistochemistry

Leaf sections (10 μm) were prepared from barley leaf pieces embedded in Steedman's wax as described by Stumpe et al. (2006) using an Automatic rotary microtome Microm HM 360 (Zeiss, Jena, Germany) with the blade lock assembly precooled to +4°C at an ambient temperature below +21°C. Single sections were placed on square coverslips of 1.5 × 1.5 cm size, coated with filtrated egg white (one section per coverslip). Sections were stretched in a drop of distilled water preheated to +40°C pipetted on the coverslips, and dried at +23°C for 2 h. Coverslips with sections were placed in Petri dishes, and dewaxing, rehydration, and blocking were performed as described by Stumpe et al. (2006). A primary anti-calreticulin antibody raised in rabbit (Baluska et al., 1999) was diluted in the ratio, 1:200 in TBS containing 1% (w/v) BSA. An anti-rabbit Alexa Fluor 488-conjugated secondary antibody produced in goat (Molecular Probes, Eugene, OR, USA) was diluted in the ratio, 1:500 in TBS with 10% BSA. In the negative controls, the primary antibody was omitted. The sections were incubated with the primary antibody for 1 h, washed three times with TBS for 15 min, incubated with the secondary antibody for 2 h, and washed three times with TBS for 10 min. After labeling, the coverslips were glued to the bottom of the inner side of Petri dishes of 35 mm diameter using melt wax as an adhesive. Prior to adhesion of sections, square hollows with a side length of ca. 1.5 cm matching the size of the coverslips had been carved into these Petri dishes with an incandescent spatula. The coverslips were put at the bottom of the Petri dishes using forceps in such a way that the sections in the Petri dish were located above the middle of the hollow. About 1 ml of glycerin was dropped into the Petri dish to cover the section. Fluorescence of Alexa Fluor 488 was examined with a Laser scanning microscope Leica TCS SP5 using 63× immersion with pZ about 276 nm and XY resolution of 150 nm. The visualization of the whole sections was performed using stereo microscope Leica TCS SP5 MP. Sections were stored at 4°C until they were processed for TEM.

Sections on the coverslips were washed three times for 5 min each in 0.1M phosphate buffer (pH 7.2-7.4) and fixed with 2.5% glutaraldehyde in the same buffer for 20 min on ice, washed again (3 × 5 min) in cold buffer and postfixed in 0.5% OsO_4_ for 10 min on ice. Sections were then washed in cold water, dehydrated in a graded ethanol series (50, 70, and 96% for 5 min each) followed by equilibration in 100% acetone, and embedded in Epon EmBed medium grade (Sigma Aldrich, MO, United States). After polymerization, coverslips were detached from the resin blocks by dipping in liquid nitrogen. Outlines of the sections were clearly visible on the surfaces of the blocks. Blocks were trimmed and series of about 140 ultrathin sections were produced. Sections were mounted on formvar coated slot grids and stained with 2% uranyl acetate and 3% lead citrate. Sections were analyzed using JEM Jeol 1400 TEM equipped with Veleta side camera (Olympus Corporation, Tokyo, Japan) at 80 kV.

### Quantification of PD and Pitfields Between Cells Using Immunofluorescence

Fluorescent puncta corresponding to single PD or pit fields containing PD-localized calreticulin were counted manually. Altogether, nine types of cell–cell boundaries were analyzed. Numbers of calreticulin-labeled PD/pitfields were counted on the following borders: (1) between anticlinal cell walls of cells of the upper epidermis; (2) between periclinal cell walls of the upper epidermal cells and of palisade parenchyma cells; (3) between anticlinal cell walls of palisade parenchyma cells; (4) between palisade parenchyma and cells of the bundle sheath of minor veins; (5) between periclinal cell walls of palisade parenchyma cells and cell walls of the adjoining spongy parenchyma cells; (6) between spongy parenchyma and cells of the bundle sheath of minor veins; (7) between spongy parenchyma cells; (8) between spongy parenchyma cells and periclinal cell walls of lower epidermis cells; (9) between anticlinal cell walls of cells of the lower epidermis. For barley leaves, mesophyll cells of the adaxial and abaxial halves of the leaf blade were analyzed separately. In all species, only minor veins of higher (4th−5th) orders were analyzed; veins of lower orders (“major” veins) were excluded from the analysis.

### Stastistical Treatment of the Data

For statistical verifications, numbers of immunolabeled PD/pitfields were counted on 10 sections per species, all sections originating from different leaves. In every section, analyses were performed in the following manner: within cells of the same tissue, where the stoichiometry of adjoining cells was typically 1:1 (epidermis/epidermis, palisade parenchyma/palisade parenchyma, spongy parenchyma/spongy parenchyma), immunolabeled PD/pitfields were counted for 10 intercellular boundaries per section (which resulted in 100 intercellular boundaries for 10 sections analyzed). Where two different tissues adjoined each other, the stoichiometry was different; e.g., in *A. meridionalis*, usually two or three palisade parenchyma cells bordered one upper epidermal cell. In these cases, the total number of immunolabeled PD/pitfields was counted on the periclinal cell walls of 10 epidermal cells bordering 20–30 parenchyma cells and normalized to one epidermal cell. This resulted in 100 epidermal cells analyzed on 10 sections per species. Similarly, the numbers of immunolabeled PD/pitfields between spongy parenchyma/palisade parenchyma, palisade parenchyma/bundle sheath, and spongy parenchyma/bundle sheath were calculated and normalized to a single cell of spongy parenchyma or palisade parenchyma, respectively. Lengths of cell walls in microns were determined on the same sections.

The significant differences in the numbers of immunolabeled PD/pitfields counted per cell surface unit was analyzed using one-way ANOVA (library “car”; Fox and Weisberg, 2019) with Tukey *post hoc* test, libraries “multcomp” (Hothorn et al., 2008) and “agricolae” (de Mendiburu, 2020). Statistical analysis and data visualization were performed using R 3.6.3 (R Studio Team, 2020) and RStudio (R Studio Team, 2020). R packages “dplyr” (Wickham et al., 2020), “ggplot2” (Wickham, 2016), “readxl” (Wickham and Bryan, 2019) and “knitr” (Xie, 2020) were used as well. For some data sets, log-transformation was applied to satisfy the assumptions of one-way ANOVA; square root transformation was used for countable data sets. The significanct differences in single cell concentrations of sugars were analyzed using Student's t-test.

## Results

### Visualization of Plasmodesmata (PD) Pitfields in Leaves of *A. barclaiana, A. meridionalis*, Barley, and Potato

To analyze the symplastic connections between cells of the leaf lamina in barley, potato, *A. barclaiana*, and *A. meridionalis*, antibodies raised against two PD-associated proteins were used, against myosin VIII (Radford and White, 1998; Reichelt et al., 1999) and calreticulin (Baluska et al., 1999; Schubert et al., 2013; Demchenko et al., 2014). Both antibodies resulted in similar labeling of PD in leaf tissues (Figure 1). In the vascular tissues, previous TEM studies had shown the presence of large PD fields at the intermediary cell/bundle sheath cell boundary in the minor veins of *A. meridionalis* leaves, and also smaller PD fields between modified intermediary cells and bundle sheath cells in the minor veins of *A. barclaiana* (Voitsekhovskaja et al., 2006). These PD fields were detected by immunofluorescence staining of calreticulin as shown in Figures 1A,B for *A. meridionalis* and *A. barclaiana*, respectively, as well as by myosin labeling (data not shown), but single PD within these fields could not be distinguished due to the small size of the cells and extremely high density of PD (Gamalei, 1991). In the parenchymatous tissues, however, PD were clearly recognized as multiple fluorescent puncta in cell walls that were sometimes seen as threads penetrating the cell walls, depending on the angle of the section (Figures 1C,D). In all the species studied, immunofluorescence revealed multiple PD between mesophyll cells (shown in Figures 1C,D for *A. meridionalis* and potato, respectively). In potato, but not in the other species, some non-specific binding of the antibodies to structures within chloroplasts occurred as can be seen in Figure 1D. This was the only non-specific labeling observed; except for this, the immunofluorescence patterns were similar to the PD-specific patterns reported in other studies with the same antibodies (Radford and White, 1998; Baluska et al., 1999; Reichelt et al., 1999; Schubert et al., 2013; Demchenko et al., 2014). Fields of fluorescent puncta like those found in the cell walls of mesophyll cells were also found between cells of neighboring epidermal cells and at the mesophyll/epidermis interface (shown in Figures 1E,F for *A. barclaiana*, Figures 1G,H for barley, Figures 1I,J for *A. meridionalis*; see also Supplementary Videos 1–8).

**Figure 2:**
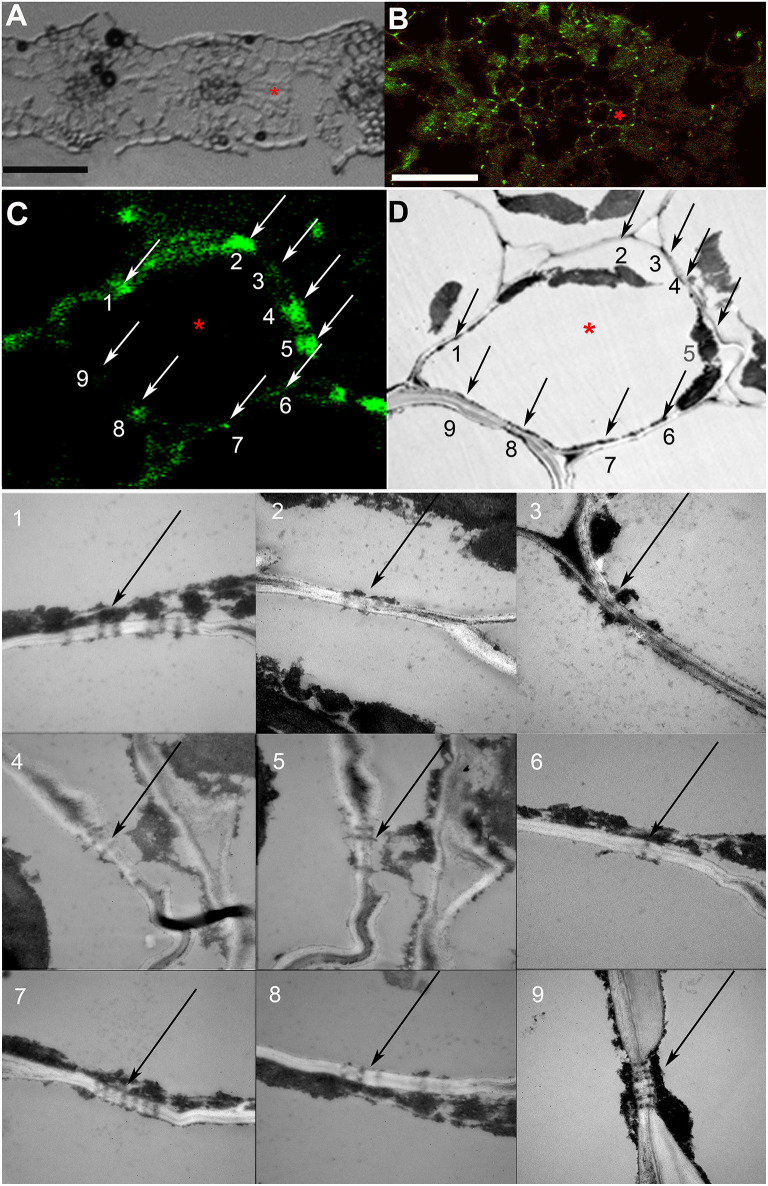
Co-localization study of immunolabeled calreticulin and of plasmodesmata (PD). **(A)** A section of a barley leaf, an asterisk marks a mesophyll cell selected for further analysis. **(B)** A single confocal laser scanning microscopy (CLSM) image of the same section, the asterisk marks the same cell as in **(A)**. **(C,D)** Immunolabeled punctate pattern in the cell walls **(C)** and TEM image **(D)** of the cell marked with asterisk in **(A)** and **(B)**. Numbered arrows **(C,D)** point at sites of the cell wall that were examined by TEM as shown in TEM images with the same numbering; black arrows point to PD and pitfields. Size bars: 50 μm **(A)**, 30 μm **(B)**.

As the resolution limit of light microscopy is ca. 200 nm, and the diameter of PD is in the range of several tenths of nanometers (Robards, 1976), several closely adjoining PD in a pitfield would be detected as a single immunolabeled fluorescent punctum. Moreover, it has been reported that in certain plant tissues, PD do not contain calreticulin (Demchenko et al., 2014). In order to address the question whether punctate pattern of immunofluorescence staining of calreticulin was associated exclusively with PD, we performed TEM analysis of immunolabeled leaf sections as shown in Figure 2 for a barley leaf. A series of ultrathin sections (70 nm) were cut through the whole depth of a semi-thin (10 μm) section which had been imaged with the confocal laser scanning microscopy (CLSM) prior to TEM (a single scan; Figures 2B,C) to visualize fluorescent puncta corresponding to PD-localized calreticulin. A cell selected for TEM analysis is marked with an asterisk in Figures 2A–D. TEM analyses revealed single, twinned, and Y-shaped PD (Figure 2, TEM images 1–9) which were grouped in pitfields containing 3–7 PD (Figure 2, arrows 1, 2, 4, 5, 7–9), or present as single PD (Figure 2, arrow 6). In the upper part of the analyzed cell, four fluorescent puncta in the cell wall (arrows 1, 2, 4, 5) corresponded to pitfields with 3–7 PD, while no PD were detected by TEM in the cell wall site without immunolabeling (arrow 3). In the lower part of the same cell, TEM study revealed a single PD (arrow 6) and three pitfields (arrows 7–9) while only sites 6 and 8 showed some immunolabeling (Figure 2C). This could be explained by a slight bending of the section that probably resulted in the lower part of the cell which is out of the CLSM focal plane. Altogether, we concluded that immunolabeled calreticulin was confined to PD in the cell walls, and that immunohistochemistry was a reliable method to detect sites of symplastic connections between leaf cells.

### Distribution of Immunolabeled PD/pitfields Between Cells and Tissues in the Leaves of *A. barclaiana, A. meridionalis*, Barley, and Potato

In order to provide a quantitative estimate of the abundance of sites of symplastic contacts between various types of cells and tissues of leaves of the four species under study, the numbers of fluorescent puncta (representing immunolabeled PD/pitfields) per length of the cell wall in microns were counted at the boundaries between all cell types except for vascular tissues. An example of a transverse section used for counting is shown in Figure 1D for the palisade cells on the left. For this procedure, we used the anti-calreticulin antibody for immunolabeling. Altogether, nine types of intercellular boundaries were analyzed. In the leaves of all species, we distinguished between upper and lower epidermis, palisade and spongy mesophyll, and cells of the bundle sheath around minor veins (major veins were excluded from the analyses). In barley leaves where only one type of mesophyll cells is present, “upper” (adaxial) and “lower” (abaxial) layers of the mesophyll were analyzed separately and are designated here as “palisade” and “spongy” to simplify the comparison with the other species. Based on leaf development, secondary PD are exclusively expected to be found at epidermis/mesophyll boundaries, while other boundaries analyzed in this study are expected to be connected by both primary and secondary PD (Satina and Blakeslee, 1941; Poethig, 1987). The numbers of immunolabeled PD/pitfields and the length of the cell walls for all cell types examined in this study are shown in Table 1.

**Table 1:** Numbers of fluorescent puncta per cell wall corresponding to anti-calreticulin-labeled PD/pitfields, and the mean lengths of shared cell walls between abutting specified cell types expressed on a per cell basis (in μm), as determined for nine types of cell/cell boundaries in leaves of *A. barclaiana, A. meridionalis*, barley, and potato.

**Type of the cell border**	***Asarina barclaiana***		***Alonsoa meridionalis***		**Potato**		**Barley**	
	**Number of PD/pitfields per cell wall**	**Length of the shared cell wall (μm)**	**Number of PD/pitfields per cell wall**	**Length of the shared cell wall (μm)**	**Number of PD/pitfields per cell wall**	**Length of the shared cell wall (μm)**	**Number of PD/pitfields per cell wall l**	**Length of the cell wall [μm]**
UE/UE	**4.4** **±** **0.5a**	*7.5 ± 1.5^*A*^*	**4.0** **±** **0.7a**	*9.2 ± 3.0^*B*^*	**0.4** **±** **0.2b**	*8.9 ± 2.2A^*B*^*	**0.2** **±** **0.1b**	*10.7 ± 2.8^*B*^*
UE/PM	**4.8** **±** **0.7a**	*13.8 ± 3.3^*A*^*	**4.0** **±** **0.5b**	*13.8 ± 4.5^*A*^*	**0.4** **±** **0.2c**	*18.6 ± 6.5^*B*^*	**0.8** **±** **0.2d**	*7.3 ± 1.6^*C*^*
PM/PM	**24.2** **±** **0.9a**	*31.0 ± 2.9^*A*^*	**14.4** **±** **1.5b**	*43.2 ± 12.6^*B*^*	**37.9** **±** **0.5c**	*86.1 ± 9.4^*C*^*	**11.1** **±** **0.5d**	*21.0 ± 6.9^*D*^*
PM/BS	**3.8** **±** **0.4a**	*12.8 ± 3.3^*AB*^*	**3.2** **±** **0.2b**	*9.9 ± 2.9^*AC*^*	**4.0** **±** **0.3a**	*15.5 ± 4.5^*B*^*	**3.5** **±** **0.3b**	*8.1 ± 2.5^*C*^*
PM/SM	**5.9** **±** **0.7a**	*11.1 ± 2.9^*A*^*	**4.1** **±** **0.3b**	*10.9 ± 2.8^*A*^*	**7.1** **±** **0.4c**	*15.3 ± 3.5^*B*^*	**5.5** **±** **0.4a**	*8.8 ± 2.5^*A*^*
SM/BS	**4.0** **±** **0.3a**	*12.8 ± 4.7^*AB*^*	**3.3** **±** **0.2b**	*9.3 ± 2.4^*AC*^*	**4.0** **±** **0.3a**	*14.5 ± 6.3^*B*^*	**4.3** **±** **0.3a**	*8.5 ± 1.4^*B*^*
SM/SM	**14.0** **±** **0.5a**	*11.3 ± 6.7^*A*^*	**10.9** **±** **0.5b**	*8.0 ± 3.5^*A*^*	**10.6** **±** **0.4b**	*11.0 ± 5.8^*A*^*	**17.3** **±** **0.6c**	*7.9 ± 3.0^*A*^*
SM/LE	**3.3** **±** **0.3a**	*11.2 ± 4.4^*A*^*	**4.2** **±** **0.3b**	*10.6 ± 2.7^*A*^*	**3.1** **±** **0.5a**	*11.1 ± 2.5^*A*^*	**1.3** **±** **0.3c**	*11.0 ± 2.7^*A*^*
LE/LE	**4.3** **±** **0.2a**	*7.1 ± 2.3^*AB*^*	**4.2** **±** **0.4a**	*8.4 ± 1.4^*AC*^*	**1.5** **±** **0.3b**	*6.3 ± 1.7^*B*^*	**1.4** **±** **0.3b**	*9.8 ± 1.6 ^*B*^*

*Data are average values for n = 10 ± SD*.

*UE, upper epidermis; PM, palisade mesophyll; BS, bundle sheath; SM, spongy mesophyll; LE, lower epidermis. Different letters indicate significant differences between species for given type of the cell border at p-value < 0.05 or less as analyzed by one-way ANOVA; lowercase letters show differences for PD/pitfields numbers per cell wall and uppercase letters for cell wall length, respectively*.

In the three dicot species analyzed, the highest numbers of fluorescent puncta per cell–cell boundary (corresponding to immunolabeled PD/pitfields) were found in the palisade mesophyll. Here, the average numbers ranged from 14 puncta per cell–cell boundary in *A. meridionalis* to 38 in potato (Table 1). A somewhat lower abundance of symplastic connections was observed for spongy parenchyma cells and between palisade and spongy parenchyma (Table 1). In the monocot barley, the highest numbers (17 puncta per cell boundary) were observed between the mesophyll cells of the abaxial part of the blade, which significantly differed from the numbers for the adaxial blade part (Table 1). In all the four species, mesophyll cells were symplastically connected to each other to the highest extent as compared to other tissues, while the lowest numbers (0.2–1.5 puncta per cell boundary) were found in leaves of barley and potato for epidermis/epidermis (upper and lower) and upper epidermis/palisade parenchyma boundaries (Table 1).

**Figure 3:**
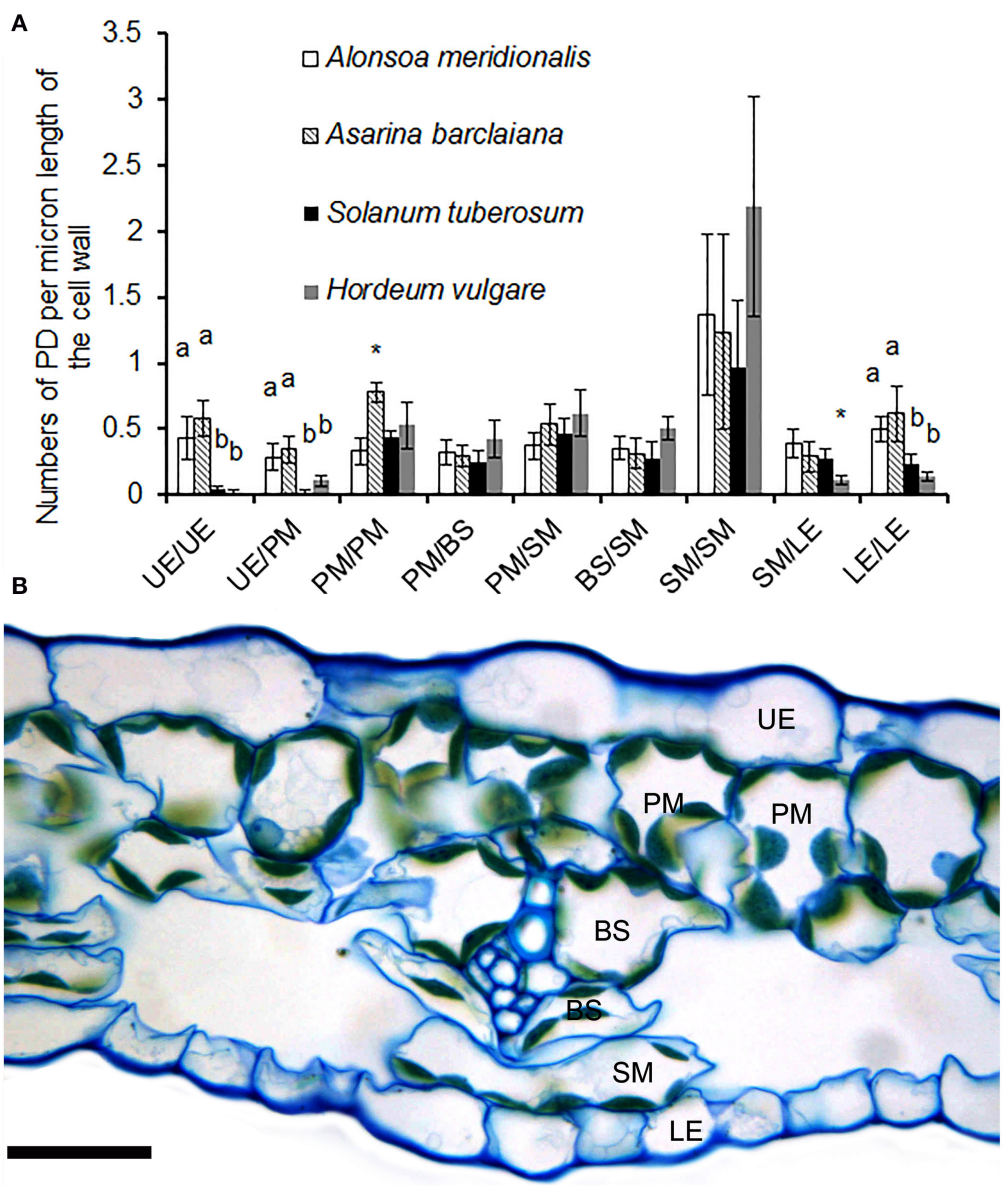
**(A)** Counts of immunolabeled PD/pitfields between different cells/tissues per μm length of cell wall as found on transverse sections through the cell wall in leaves of *A. meridionalis, A. barclaiana*, potato (*Solanum tuberosum*), and barley (*Hordeum vulgare*). Data represent average values for 10 cell borders ± SD. UE, upper epidermis; PM, palisade mesophyll; BS, bundle sheath; SM, spongy mesophyll; LE, lower epidermis. Different letters indicate significant differences between species for a boundary type at least at the level of *p* < 0.01, * stands for significant differences at the level of *p* < 0.05, according to one-way ANOVA with *post hoc* Tukey's test. **(B)** Cross-section of a leaf of *A. barclaiana* showing the position of the analyzed cell types within the leaf lamina. Size bar: 20 μm.

When the lengths of the cell walls were taken into account, the highest values (1–2 fluorescent puncta corresponding to immunolabeled PD/pitfields per micron of cell wall length) were observed between cells of the spongy parenchyma in all species (Figure 3A). A cross-section of the leaf of *A. barclaiana* indicating the position of the analyzed cell types within the leaf lamina is shown in Figure 3B. The values of other cell types were much lower (0.25–0.60 immunolabeled PD/pitfields μm^−1^ CW length). Strikingly low symplastic connectivity (0.02–0.10 immunolabeled PD/pitfields μm^−1^ CW length) was found for upper epidermis/upper epidermis and upper epidermis/palisade mesophyll boundaries in barley and potato, as well as for lower epidermis/lower epidermis and lower epidermis/spongy mesophyll boundaries in barley. At the same time, values for boundaries of epidermal cells in *A. meridionalis* and *A. barclaiana* did not differ from those for other cell types, being in the range of 0.3–0.6 immunolabeled PD/pitfields μm^−1^ CW length (Figure 3A).

### Distribution of PD in Shoot Apical Meristems of *A. barclaiana, A. meridionalis*, Barley, and Potato

**Figure 4:**
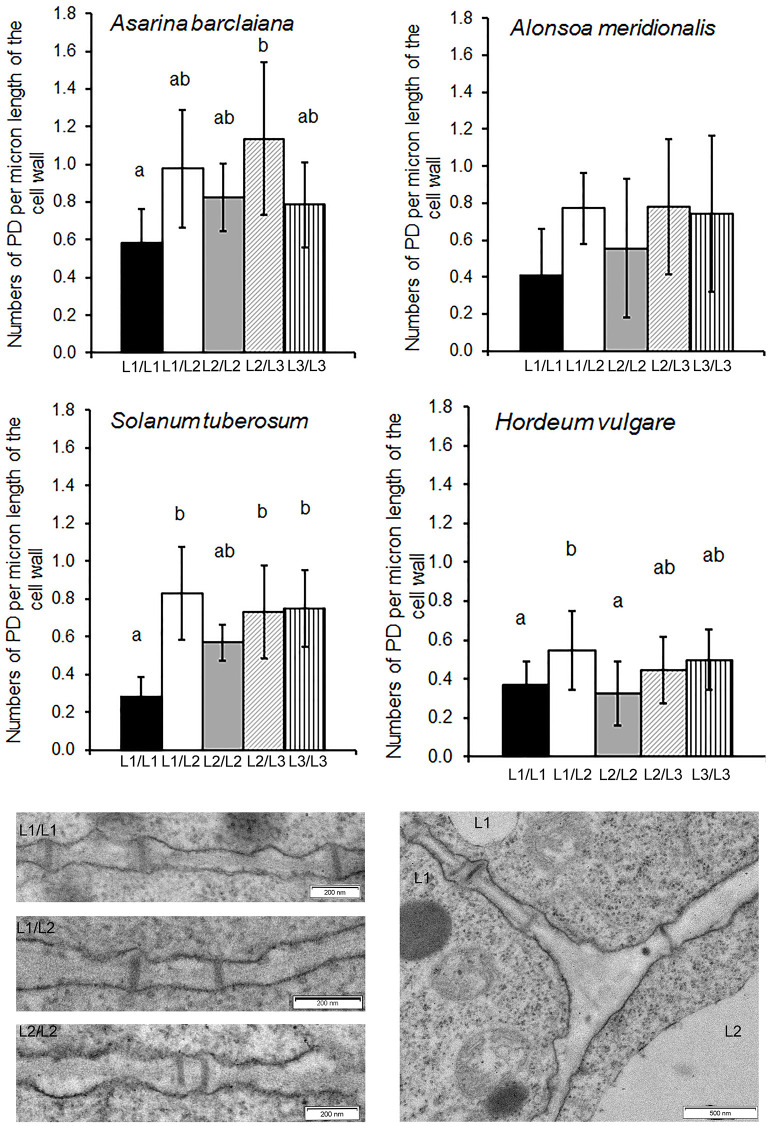
Quantitative estimates for PD between different cells/layers in shoot apical meristems of *A. meridionalis, A. barclaiana*, potato, and barley. PD frequencies are expressed as numbers of PD per μm length of cell wall as found on transverse sections through the cell wall. Data represent average values for 6–25 cells ± SE. Different letters indicate significant differences at *p* < 0.05 according to one-way ANOVA with *post hoc* Tukey's test. Data for barley are reproduced from Dmitrieva et al. (2017). Micrographs show PD in one of the *Solanum tuberosum* apices examined. L1 and L2 mark cells of the corresponding cell layers.

In angiosperms, shoot apical meristems (SAMs) are organized in distinct cell layers originating from the initial cells. Cells of the L1 and sometimes L2 layers, forming the tunica, undergo anticlinal divisions while cells of the L3 layer do not show a distinct pattern of divisions (see also Supplementary Figure 1 for L1 in *A. barclaiana* and *A. meridionalis* SAMs). Based on this model, it is easy to conclude that secondary PD are present at L1/L2 and at L2/L3 boundaries, and primary PD connect cells of the same layer (although the formation of secondary PD between cells derived from the same layer is possible; Fitzgibbon et al., 2013). PD at the borders of cells belonging to L1 and L2 layers, as well as at L1/L2 and L2/L3 boundaries, were quantified in SAMs of *A. barclaiana, A. meridionalis*, barley, and potato by means of TEM, because the small size of the SAM cells would not allow reliable determinations using immunohistochemistry as was performed for leaves. The patterns of PD distribution and their frequencies were generally similar in the SAMs of all species studied (Figure 4). The frequencies of PD between cells of the L1 layer (designated as primary PD) were significantly lower than the frequencies of PD between cells of L1 and L2 (secondary PD) in potato and barley, but not in the other species, at the level of *p* < 0.05. A general tendency to form more secondary PD than primary PD per cell wall length unit was apparent for all species.

### Concentrations of Sucrose and Hexoses in Epidermal and Mesophyll Cells of *A. meridionalis* and *A. barclaiana*

The level of symplastic connectivity between epidermis and mesophyll in *A. meridionalis* and *A. barclaiana* was similar to that between mesophyll cells, in contrast to barley and potato, where symplastic connectivity was higher within the mesophyll than between the epidermis and mesophyll (Figure 3A). Thus, the question arose whether symplastic exchange of sugars between epidermis and mesophyll might occur in *A. meridionalis* and *A. barclaiana*. We therefore analyzed the concentrations of sucrose and hexoses in single epidermal and mesophyll cells in *A. meridionalis* and *A. barclaiana* during the day and during forced accumulation of sugars in leaves due to blockage of export *via* the phloem.

**Figure 5:**
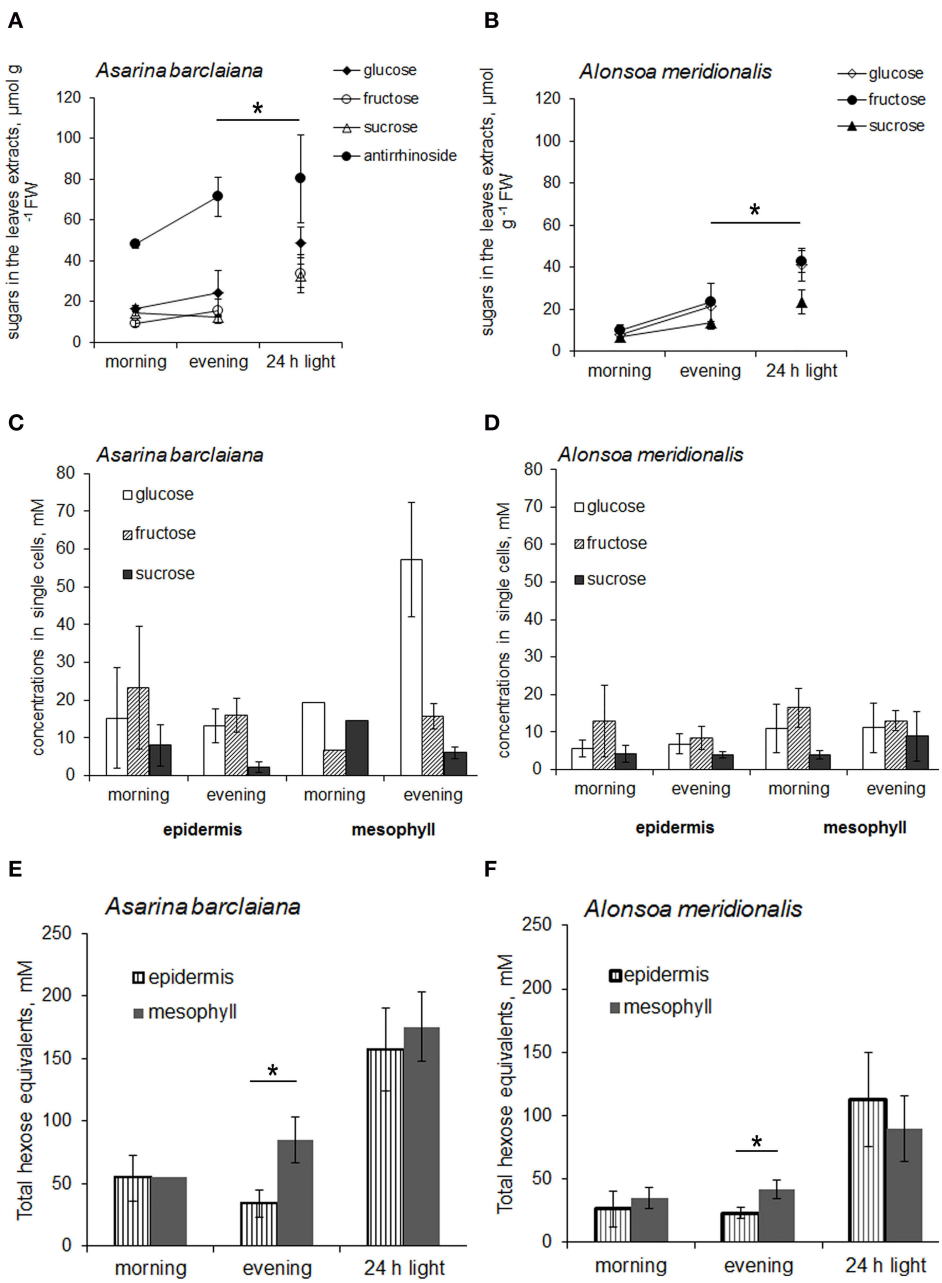
Sugar concentrations in leaves **(A,B)** and in single cells samples obtained from the epidermis and mesophyll **(C–F)** of *A. barclaiana* **(A,C,E)** and *A. meridionalis* **(B,D,F)** at the beginning of the light period after 3 h of illumination (“morning”), at the end of the light period after 11 h illumination (“evening”), and after 24 h exposure of detached leaves to continuous light, respectively. **(A,B)** Open triangles stand for glucose, closed triangles for fructose, open circles for sucrose, and closed circles for antirrhinoside, respectively. Mean values of 3–5 independent measurements ± SE are shown except for the “morning” time points in **(C)** and **(E)** which represent the values from a single measurement. Data in **(E)** and **(F)** are expressed on the basis of hexose equivalents for total amounts of glucose, fructose, and sucrose. Asterisks indicate significant different values at least at the *p* < 0.05 level according to Student's *t*-test, except for antirrhinoside contents **(A)** where differences between “evening” and “24 h light” points were non-significant.

First, the levels of non-structural carbohydrates were measured in the whole leaves of *A. barclaiana* and *A. meridionalis*, and no pronounced changes were observed over the diurnal rhythm (Figures 5A,B). In order to cause the accumulation of soluble carbohydrates in the leaves, leaves were detached from the plants after 3 h of the light period and placed in continuous light for 24 h, while the petioles were kept in a 2 mM CaCl_2_ solution to favor sealing of the phloem with callose (King and Zeevaart, 1974). This treatment disrupted sugar export from the leaves while photosynthesis continued. At the end of the light exposure period, concentrations of sucrose, glucose, and fructose had increased significantly in the detached leaves from *A. barclaiana* and *A. meridionalis*, as compared to their levels at the end of the day in normally functioning attached leaves (Figures 5A,B). Another major carbohydrate-conjugated compound, the iridoid glucoside antirrhinoside, found in *A. barclaiana* (Voitsekhovskaja et al., 2006), showed no significant accumulation upon export blockage from the leaves (Figure 5A); neither did mannitol, myo-inositol, galactinol, raffinose, and stachyose in leaves of both species (data not shown).

The concentrations of glucose, fructose, and sucrose in the epidermal cells of *A. meridionalis* and *A. barclaiana* were compared with those in mesophyll cells in the morning (after 3 h of illumination) and in the evening (after 11 h of illumination) at the single-cell level in plants growing under a 16 h light/8 h dark regime. The results are shown in Figures 5C,D. At both “morning” and “evening” time points, the levels of these sugars were in a similar range for epidermis and mesophyll cells in both species, except for the accumulation of glucose in mesophyll cells of *A. barclaiana* in the “evening”. When expressed on the basis of total hexose units, the “morning” concentrations of soluble sugars in mesophyll cells did not differ significantly from those in epidermal cells in these species and were in the range between 20 and 70 mM (Figures 5E,F). At the “evening” time points, sugar concentrations in mesophyll cells of both species were in the range between 20 and 100 mM and thus were significantly higher than those in epidermal cells (20–70 mM) at the level of *p* < 0.05 (Figures 5E,F).

When the assimilate export from the leaves was blocked by the detachment of the leaves, the epidermal cells in both plants accumulated soluble carbohydrates up to the levels measured in mesophyll cells as shown by the concentrations of sucrose and hexoses measured in the single cells of detached leaves after 24 h of illumination (Figures 5E,F). In *A. barclaiana*, the single-cell total sugar concentrations (calculated as a sum of hexoses and sucrose, expressed in hexose units) were 157 ± 40 mM in the epidermal cells and 175 ± 50 mM in the mesophyll cells. In *A. meridionalis*, these values were 126 ± 50 mM in epidermal cells and 113 ± 40 mM in mesophyll cells. These data indicate that in *A. meridionalis* and *A. barclaiana* leaves, the epidermis is able to accumulate soluble sugars.

## Discussion

Determination of cell fate and specification of cell layers in SAMs as well as in leaves during their development requires precise regulation of symplastic exchange between cells. A whole orchestra of regulators, such as transcription factors, RNA, and small molecules like hormones move through PD enabling SAM functioning as well as proper development of leaves from leaf primordia (Kitagawa and Jackson, 2017; Liu and Chen, 2018; Bhatia et al., 2021; Maksimova et al., 2021; Romanova et al., 2021). Moreover, also in the mature leaves, exchange of regulatory molecules as well as assimilates between cells and tissues *via* PD is a highly important and finely tuned process (Cui et al., 2014; Liu and Chen, 2018; Dmitrieva et al., 2021). Recent studies further reveal the role of metabolite networks in the regulation of the development (Omidbakhshfard et al., 2020). To understand the whole-leaf compartmentation of developmental regulators as well as metabolites, mapping of symplastic connections and domains within plant organs, and our understanding of how they emerge and change during development, is of high importance.

The establishment of primary PD during leaf development occurs between cells of the same lineage, that is, cells that are derived from a single mother cell and share a common division cell wall. Such lineages can be easily observed, and the fate of their cells traced, within SAMs; in angiosperms, they include the superficial cell layer L1, the subsuperficial layer L2, and the bulk of meristematic cells in the center of SAMs designated as layer L3 (Kang and Dengler, 2018). PD at L1/L2 and L2/L3 boundaries are obligatorily secondary (Evkaikina et al., 2014). Similarly, leaf primordia in angiosperms include layers, L1, L2, and L3 that produce the epidermis, the leaf parenchyma, and the central vascular bundle, respectively (Satina and Blakeslee, 1941; Sharman, 1945; Poethig, 1987; Alvarez et al., 2016; Du et al., 2018). However, as a leaf develops *via* the action of marginal and plate meristems, tracing cell lineages originating from these layers becomes challenging (Poethig, 1987; Alvarez et al., 2016; Du et al., 2018). Nevertheless, studies on periclinal chlorophyll chimeras and colchicine-induced cytochimeras performed on the monocot and dicot species revealed that the leaf epidermis always originates from L1, the palisade mesophyll is produced by L2, the spongy mesophyll (including the leaf minor veins) originates from L2 and/or L3, and the main conducting bundle of the leaf is produced by L3 (Dermen, 1947, 1953; Stewart and Burk, 1970; Poethig, 1987). Within L2-derivatives, minor veins and bundle sheath cells originate from a different cell lineage than the mesophyll in the C4 grass, *Stenotaphrum secundatum* (Sud and Dengler, 2000), while in barley, bundle sheath cells and mesophylls originate from another lineage than mestome sheath and vascular tissues (both major and minor veins) (Trivett and Evert, 1998). In dicots, the minor veins originate from the same cell lineage as the spongy mesophyll (Dermen, 1947, 1951).

In this study, the question was asked whether in plants with highly developed secondary PD at the mesophyll/phloem interface, such as the symplastic phloem loaders, *Asarina barclaiana* and *Alonsoa meridionalis*, secondary PD formation is generally enhanced at other boundaries, contrary to apoplastic phloem loaders which in our study were represented by potato and barley. According to the above mentioned studies, secondary PD are present at L1/L2 and L2/L3 boundaries in SAMs, at the epidermis/mesophyll interface in leaves, and they can be expected to occur in leaves between palisade mesophyll and spongy mesophyll, and between palisade mesophyll and the bundle sheath of minor veins. The TEM of SAMs did not reveal any differences in the secondary PD formation between the species under examination (Figure 4); rather, a general tendency to form more secondary PD than primary PD was apparent for all species. To obtain a quantitative estimation of the distribution of PD between different cell types within leaf laminae, immunostaining with antibodies against a PD-specific protein was used (Danila et al., 2016). We applied an antibody raised against calreticulin, which is a major chaperone protein in the lumen of the endoplasmic reticulum (ER); however, it is also shown to represent a reliable PD marker in different plant species and tissues, such as maize root apices (Baluska et al., 1999) and symbiotic root nodules of *Casuarina glauca* (Demchenko et al., 2014), respectively. Baluska et al. (1999) showed that calreticulin is located at the ER domain of the PD, and not at other ER compartments. This is in contrast to another ER-specific protein, calnexin, which was found both in ER strands located in the cytoplasm and in PD in Arabidopsis (Liu et al., 2015).

In our study, in the leaves of the four species, labeling was highly specific to immunolabeled PD/pitfields in the cell walls (Figure 1; Supplementary Videos 1–8). This specificity was further confirmed by TEM studies of immunolabeled leaf sections (Figure 2); this approach was taken as a substitute for correlation light electron microscopy (CLEM) (Brault et al., 2019; Modla et al., 2020), because the latter is based on the expression of genes encoding PD-targeted fluorescent proteins that was not possible for the species used in this study. Interestingly, although the data confirmed that a single fluorescent punctum corresponded in most cases to pitfields with multiple PD (Figure 2), estimates of the numbers of puncta for barley and potato were similar to or slightly higher than those determined using TEM analyses at the same tissue boundaries which confidently distinguished separate PD (Table 2). Thus, PD labeling by means of calreticulin antibodies seems to produce reliable estimates of the numbers of sites of symplastic connections within the leaf epidermis and parenchyma, although it cannot be applied to tissues where PD numbers are much higher, but to the smaller cells, than parenchyma cells, e.g., to the phloem (Figures 1A,B).

**Table 2:** Comparison of the numbers of immunolabeled PD/pitfields with PD frequencies (PD μm^−1^ length CW) determined in leaves of several species by means of immunolabeling (this study) and TEM (literature data), respectively.

**Cell**	***Coleus blumei*^**A**^**	***Alonsoa meridionalis*^**B**^**	***Hordeum vulgare***		***Solanum tuberosum***	
**boundary**	**(TEM)**	**(Immunolabeling)**	***(TEM and immunolabeling)***		***(TEM and immunolabeling)***	
			**TEM**	**Immuno-labeling**	**TEM^**D**^**	**Immuno-labeling^**B**^**
UE/UE	0.19	0.43	–	–	–	–
UE/PM	–	–	0.05^E^	–	0.01	0.02
PM/PM	0.19	0.33	0.13^E^	0.1^E^	0.05	0.44
PM/BS	0.12	0.32	0.25^C^	0.42^B^	0.09	0.26
PM/SM	0.10	0.37	–	–	–	–
SM/BS	0.18	0.35	–	–	0.13	0.28
SM/SM	0.26	1.37	–	–	0.15	0.96
LE/SM	–	–	–	–	0.05	0.28

*UE, upper epidermis; PM, palisade mesophyll; BS, bundle sheath; SM, spongy mesophyll; LE, lower epidermis. “-” stands for variants where no TEM data are available for comparison*.

A *Data from Fisher (1986). Coleus blumei belongs to the Lamiaceae family and is a close relative of A. meridionalis from the Scrophulariaceae family, both from the order Lamiales. Both species possess phloem companion cells of the intermediary cell type with similar abundance of secondary PD*.

B *This study*.

C *Data from Evert et al. (1996)*.

D *Data from McCauley and Evert (1989)*.

E *Data from Dmitrieva et al. (2017)*.

In both symplastic and apoplastic phloem loaders analyzed in the present study, numbers of immunolabeled PD/pitfields at cell boundaries other than bundle sheath/phloem companion cells did not show any correlation with the loading mode, independent of whether the boundaries contained primary or secondary PD. This was observed for both leaves and SAMs (Figures 3, 4), with only one exception found in leaf laminae. The numbers of immunolabeled PD/pitfields at the epidermis/epidermis and epidermis/mesophyll boundaries were strikingly low in the apoplastic loaders, potato and barley, while in the symplastic loaders, *A. meridionalis* and *A. barclaiana*, these numbers did not differ much from those between mesophyll cells (Figure 3).

Remarkably, the numbers of immunolabeled PD/pitfieldsper length unit of cell walls between spongy parenchyma cells in the dicot species, *A. meridionalis, A. barclaiana*, and potato, as well as between mesophyll cells of the abaxial part of the leaf blade in the monocot barley, were significantly higher than those numbers at other interfaces in leaf laminae (Figure 3). In dicot leaves, spongy mesophyll cells are surrounded by large intercellular spaces due to the abaxial position of the stomata, so that the metabolite exchange *via* apoplastic water phase is probably restricted, necessitating the compensatory enhancement of symplastic traffic. However, stomata and intercellular spaces are located at both abaxial and adaxial sides in barley leaves. Therefore, a more plausible explanation for the increase in PD frequencies in the abaxial mesophyll might be the proximity of the cells to the phloem part of the leaf veins which are abaxially positioned in dicots as well as monocots. Movement of sugars from the mesophyll to the phloem occurs *via* the symplast through PD in the leaf lamina. The reason for this is probably the necessity to compartmentalize the sugar flux in order not to be perturbed by the transpiration water flow, which has the opposite direction and is compartmentalized to the apoplast. Thus an increase of PD frequency on the abaxial side of the leaf lamina might reflect the intensification of symplastic transport near the phloem. However, more studies are required to confirm this interpretation.

It is well known that the vacuoles of mesophyll cells can serve as a temporary storage compartment for sucrose and sucrose-derived hexoses, in order to avoid their accumulation in the cytosol which could cause osmotic swelling of the cytoplasm and lead to disturbances in cytosolic metabolism (Heber and Kaiser, 1984). Usually, the cells with the strongest vacuolization are those of the leaf epidermis (Winter et al., 1993, 1994; Leidreiter et al., 1995), which theoretically could provide an additional temporal sink for an excess of soluble sugars in leaves. However, earlier studies on barley and potato, where sugar contents were measured in individual mesophyll and epidermal cells, led to the conclusion that the epidermis does not take part in the partitioning of soluble sugars within leaves. In barley, the levels of sucrose and hexoses in the epidermal cells were negligible throughout the day as well as under conditions of inhibited export of assimilates from the leaves, when malate is known to accumulate in the epidermis (Koroleva et al., 1997, 1998, 2000). In wild-type potato plants, the levels of soluble sugars in the epidermis were very low as compared to the mesophyll, although high amounts of sucrose and hexoses accumulated in epidermal cells of transgenic plants with severely impaired phloem loading (Kehr et al., 1998, 1999). Yet, in the symplastic loaders, such as *Asarina barclaiana* and *Alonsoa meridionalis*, the epidermal cells of leaf accumulated soluble sugars up to levels similar to those of mesophyll cells. We assume that these sugars entered the epidermis *via* the PD connecting both tissues.

Accumulation of sugars in the leaf epidermis of symplastic loaders as found in the present study might have consequences for the regulation of guard cells by apoplastic sucrose. In apoplastic phloem loaders, apoplastic sucrose supplied to guard cells together with abscisic acid (ABA) from transpiration stream promoted guard cell closure (Kang et al., 2007a; Daloso et al., 2016; Antunes et al., 2017). It was hypothesized that the apoplastic sucrose concentration around guard cells represents an integrating signal allowing guard cells to keep pace with transpiration, photosynthesis, and phloem translocation rates. However, this regulation of stomata by apoplastic sucrose levels was lacking in symplastic loaders (Kang et al., 2007b). Accumulation of sucrose in the epidermis of the leaves of symplastic phloem loaders as shown in the present study might provide an alternative mechanism of the regulation of stomata in the absence of the necessary levels of apoplastic sucrose, not related to the uptake of sucrose from the apoplast into guard cells (Antunes et al., 2017).

In conclusion, the estimation of symplastic connectivity in leaf laminae, as well as between cells in SAMs, analyzed in this study for four species differing in their phloem loading mode, did not reveal a general increase of the formation of secondary PD in symplastic relative to apoplastic phloem loaders. In the two symplastic loaders, the leaf epidermis was shown to be able to accumulate sugars to levels similar to those found in mesophyll cells. The species, *A. barclaiana* and *A. meridionalis*, contained on average from 3 to 15 times more immunolabeled PD/pitfields per length cell wall between epidermal and mesophyll cells than barley and potato which do not accumulate sugars in the epidermis. This suggests that in the symplastic phloem loaders, the exchange of sugars between the mesophyll and the epidermis can occur *via* the symplastic pathway, rather than the apoplastic pathway, and that in these species, the leaf epidermis forms a symplastic domain together with the mesophyll. This is a novel role of the leaf epidermis, and it would be interesting to gain more information on the occurrence of this phenomenon as well as on how it might influence other functions of the epidermis, such as its roles as barrier for pathogens and in the regulation of stomatal closure.

## Data Availability Statement

The raw data supporting the conclusions of this article will be made available by the authors, without undue reservation.

## Author Contributions

OV and OK conceptualized the research and performed single cell sampling. AT and GL supervised the research. OV performed sugar analyses and wrote the manuscript. ANM and AIM performed morphometric analyses. KD performed immunolocalization of myosin VIII and calreticulin in PD. VD performed immunolocalization of calreticulin on leaf sections used for TEM. AI performed TEM studies. ET and OV analyzed TEM data. All authors analyzed and discussed the data including the production of figures. All authors read and approved the final version of the article.

## Conflict of Interest

The authors declare that the research was conducted in the absence of any commercial or financial relationships that could be construed as a potential conflict of interest.

## Publisher's Note

All claims expressed in this article are solely those of the authors and do not necessarily represent those of their affiliated organizations, or those of the publisher, the editors and the reviewers. Any product that may be evaluated in this article, or claim that may be made by its manufacturer, is not guaranteed or endorsed by the publisher.

## Supplementary Material

Supplementary Figure 1 Sections of shoot apices of *Asarina barclaiana* **(A)** and *Alonsoa meridionalis* **(B)**. Within the shoot apical meristems, the tunica consists of the L1 layer which is continuous with the protoderm layer of leaf primordia (P) as outlined in red. Arrows point on glandular hairs. Shoot apices were embedded in epon resin and semi-thin sections were produced as described in Materials and Methods. The sections were stained with toluidine blue (0.05%). Scale bar: 20 μm.

Supplementary Video 1 Plasmodesmata (PD) between cells of the palisade parenchyma in leaves of *Asarina barclaiana* (arrows point at selected face-on views) as visualized by means of immunohistochemistry using calreticulin-specific antibodies.

Supplementary Video 2 Plasmodesmata (PD) between cells of the spongy parenchyma in leaves of *Asarina barclaiana* (arrows point at selected face-on views) as visualized by means of immunohistochemistry using calreticulin-specific antibodies.

Supplementary Video 3 Plasmodesmata (PD) between cells of the palisade parenchyma in leaves of *Alonsoa meridionalis* (arrows point at selected face-on views) as visualized by means of immunohistochemistry using calreticulin-specific antibodies.

Supplementary Video 4 Plasmodesmata (PD) between cells of the palisade parenchyma in leaves of *Alonsoa meridionalis* (arrows point at selected face-on views) as visualized by means of immunohistochemistry using calreticulin-specific antibodies.

Supplementary Video 5 Plasmodesmata (PD) between cells of the palisade parenchyma in leaves of *Solanum tuberosum* (arrows point at selected face-on views) as visualized by means of immunohistochemistry using calreticulin-specific antibodies.

Supplementary Video 6 Plasmodesmata (PD) between cells of the spongy parenchyma (top; arrows point at selected face-on views) and between the cells of the palisade parenchyma and the bundle sheath (a single arrow; bottom) in leaves of *Solanum tuberosum*. In the center, a xylem ending surrounded by bundle sheath cells is shown. The PD were visualized by means of immunohistochemistry using calreticulin-specific antibodies; note unspecific staining of starch grains in this species.

Supplementary Video 7 A section through a leaf of *Hordeum vulgare* showing plasmodesmata (PD) between mesophyll cells in the adaxial and abaxial parts of the leaf (arrows). The PD were visualized by means of immunohistochemistry using calreticulin-specific antibodies.

Supplementary Video 8 Plasmodesmata (PD) between cells of the spongy parenchyma in leaves of *Hordeum vulgare* (arrow points at face-on view) as visualized by means of immunohistochemistry using calreticulin-specific antibodies.

## References

[B1] Alvarez J. P. Furumizu C. Efroni I. Eshed Y. Bowman J. L. (2016). Active suppression of a leaf meristem orchestrates determinate leaf growth. eLife 5:e15023. 10.7554/eLife.15023 27710768 PMC5096885

[B2] Antunes W. C. Daloso D. M. Pinheiro D. P. Williams T. C. R. Loureiro M. E. (2017). Guard cell-specific down-regulation of the sucrose transporter SUT1 leads to improved water use efficiency and reveals the interplay between carbohydrate metabolism and K+ accumulation in the regulation of stomatal opening. Environ. Exp. Bot. 135, 73–85. 10.1016/j.envexpbot.2016.12.004

[B3] Baluska F. Samaj J. Napier R. Volkmann D. (1999). Maize calreticulin localizes preferentially to plasmodesmata in root apex. Plant J. 19, 481–488. 10.1046/j.1365-313X.1999.00530.x 10504570

[B4] Batashev D. R. Pakhomova M. V. Razumovskaya A. V. Voitsekhovskaja O. V. Gamalei Y. V. (2013). Cytology of the minor-vein phloem in 320 species from the subclass Asteridae suggests a high diversity of phloem-loading modes. Front. Plant Sci. 4:312. 10.3389/fpls.2013.00312 23970890 PMC3748319

[B5] Bhatia S. Kumar H. Mahajan M. Yadav S. Saini P. Yadav S.. (2021). A cellular expression map of epidermal and subepidermal cell layer-enriched transcription factor genes integrated with the regulatory network in Arabidopsis shoot apical meristem. Plant Direct. 5:e00306. 10.1002/pld3.306 33748654 PMC7970154

[B6] Botha C. E. J. Cross R. H. M. (1997).Plasmodesmatal frequency in relation to short-distance transport and phloem loading in leaves of barley (*Hordeum vulgare*). Phloem is not loaded directly from the symplast. Physiol. Plant. 99, 355–362. 10.1034/j.1399-3054.1997.990301.x

[B7] Brault M. L. Petit J. D. Immel F. Nicolas W. J. Glavier M. Brocard L.. (2019). Multiple C2 domains and transmembrane region proteins (MCTPs) tether membranes at plasmodesmata. EMBO Rep. 20:e47182. 10.15252/embr.201847182 31286648 PMC6680132

[B8] Cui H. Kong D. Liu X. Hao Y. (2014). SCARECROW, SCR-LIKE 23 and SHORT-ROOT control bundle sheath cell fate and function in *Arabidopsis thaliana*. Plant J. 78, 319–327. 10.1111/tpj.12470 24517883

[B9] Daloso D. M. dos Anjos L. Fernie A. R. (2016). Roles of sucrose in guard cell regulation. New Phytol. 211, 809–818. 10.1111/nph.13950 27060199

[B10] Danila F. R. Quick W. P. White R. G. Furbank R. T. von Caemmerer S. (2016). The metabolite pathway between bundle sheath and mesophyll: quantification of plasmodesmata in leaves of C3 and C4 monocots. Plant Cell 28, 1461–1471. 10.1105/tpc.16.00155 27288224 PMC4944413

[B11] de Mendiburu F. (2020). agricolae: Statistical Procedures for Agricultural Research. R package version 1.3-3. Available online at: https://CRAN.R-project.org/package=agricolae (accessed March 29, 2021).

[B12] Demchenko K. N. Voitsekhovskaja O. V. Pawlowski K. (2014). Plasmodesmata without callose and calreticulin in higher plants—open channels for fast symplastic transport? Front. Plant Sci. 5:74. 10.3389/fpls.2014.00074 24634671 PMC3943419

[B13] Dermen H. (1947). Periclinal cytochimeras and histogenesis in cranberry. Am. J. Bot. 34, 32–43. 10.1002/j.1537-2197.1947.tb12955.x

[B14] Dermen H. (1951). Ontogeny of tissues in stem and leaf of cytochimeral apples. Am. J. Bot. 38, 753–760. 10.1002/j.1537-2197.1951.tb14888.x

[B15] Dermen H. (1953). Periclinal cytochimeras and origin of tissues in stem and leaf of peach. Am. J. Bot. 40, 154–168. 10.1002/j.1537-2197.1953.tb06463.x

[B16] Dmitrieva V. A. Domashkina V. V. Ivanova A. N. Sukhov V. S. Tyutereva E. V. Voitsekhovskaja O. V. (2021). Regulation of plasmodesmata in leaves of *Arabidopsis*: ATP, NADPH and chlorophyll *b* levels matter. J. Exp. Bot. (inpress). 10.1093/jxb/erab205 33974689

[B17] Dmitrieva V. A. Ivanova A. N. Tyutereva E. V. Evkaikina A. I. Klimova E. A. Voitsekhovskaja O. V. (2017). Chlorophyllide-a-Oxygenase (CAO) deficiency affects the levels of singlet oxygen and formation of plasmodesmata in leaves and shoot apical meristems of barley. Plant Signal. Behav. 12:e1300732. 10.1080/15592324.2017.1300732 28272988 PMC5437820

[B18] Du F. Guan C. Jiao Y. (2018). Molecular mechanisms of leaf morphogenesis. Mol. Plant 11, 1117–1134. 10.1016/j.molp.2018.06.006 29960106

[B19] Evert R. F. Russin W. A. Botha C. E. J. (1996). Distribution and frequency of plasmodesmata in relation to photoassimilate pathway and phloem loading in the barley leaf. Planta 198, 572–579. 10.1007/BF00262644 28321668

[B20] Evkaikina A. I. Romanova M. A. Voitsekhovskaja O. V. (2014). Evolutionary aspects of non-cell-autonomous regulation in vascular plants: structural background and models to study. Front. Plant Sci. 5:31. 10.3389/fpls.2014.00031 24575105 PMC3920070

[B21] Fisher D. G. (1986). Ultrastructure, plasmodesmatal frequency, and solute concentration in green areas of variegated *Coleus blumei* Benth. leaves. Planta 169, 141–152. 10.1007/BF00392308 24232544

[B22] Fitzgibbon J. Beck M. Zhou J. Faulkner C. Robatzek S. Oparka K. (2013). A developmental framework for complex plasmodesmata formation revealed by large-scale imaging of the Arabidopsis leaf epidermis. Plant Cell 21, 57–70. 10.1105/tpc.112.105890 23371949 PMC3584549

[B23] Fox J. Weisberg S. (2019). An R Companion to Applied Regression, Third Edition. Thousand Oaks, CA: Sage.

[B24] Gamalei Y. V. (1991). Phloem loading and its development related to plant evolution from trees to herbs. Trees 5, 50–64. 10.1007/BF00225335

[B25] Gamalei Y. V. (1995). Comparative biology of trees and herbs: intercellular communication, in L'Arbre. Biologie et Development - 3ème colloque, ed Edelin C. (Montpellier), 1–11.

[B26] Heber U. Kaiser G. (1984). Sucrose transport into vacuoles isolated from barley mesophyll protoplasts. Planta 161, 562–568. 10.1007/BF00407090 24253927

[B27] Hothorn T. Bretz F. Westfall P. (2008). Simultaneous inference in general parametric models. Biomet. J. 50, 346–363. 10.1002/bimj.200810425 18481363

[B28] Kang J. Dengler N. G. (2018). Leaf architecture: regulation of leaf position, shape and internal structure, in Annual Plant Reviews Online, eds Roberts J. A.. 10.1002/9781119312994.apr0164

[B29] Kang Y. Outlaw W. H. Jr Andersen P. C. Fiore G. B. (2007a). Guard cell apoplastic sucrose concentration—a link between leaf photosynthesis and stomatal aperture size in the apoplastic phloem loader *Vicia faba* L. Plant Cell Environ. 30, 551–558. 10.1111/j.1365-3040.2007.01635.x 17407533

[B30] Kang Y. Outlaw W. H. Jr Fiore G. B. Riddle K. A. (2007b). Guard cell apoplastic photosynthate accumulation corresponds to a phloem-loading mechanism. J. Exp. Bot. 58, 4061–4070. 10.1093/jxb/erm262 18182421

[B31] Kehr J. Hustiak F. Walz C. Willmitzer L. Fisahn J. (1998). Transgenic plants changed in carbon allocation pattern display a shift in diurnal growth pattern. Plant J. 16, 497–503. 10.1046/j.1365-313x.1998.00318.x 9881169

[B32] Kehr J. Wagner U. Willmitzer L. Fisahn J. (1999). Effect of modified carbon allocation on turgor, osmolality, sugar and potassium content, and membrane potential in the epidermis of transgenic potato (*Solanum tuberosum* L.) plants. J. Exp. Bot. 50, 565–571. 10.1093/jxb/50.334.565

[B33] King R. W. Zeevaart J. A. D. (1974). Enhancement of phloem exudation from cut petioles by chelating agents. Plant Physiol. 53, 96–103. 10.1104/pp.53.1.96 16658661 PMC541341

[B34] Kitagawa M. Jackson D. (2017). Plasmodesmata-mediated cell-to-cell communication in the shoot apical meristem: how stem cells talk. Plants 6:12. 10.3390/plants6010012 28257070 PMC5371771

[B35] Koroleva O. A. Farrar J. F. Tomos A. D. Pollock C. J. (1997). Patterns of solute in individual mesophyll, bundle sheath and epidermal cells of barley leaves induced to accumulate carbohydrate. New Phytol. 136, 97–104. 10.1111/j.1469-8137.1997.tb04735.x

[B36] Koroleva O. A. Farrar J. F. Tomos A. D. Pollock C. J. (1998). Carbohydrates in individual cells of epidermis, mesophyll, and bundle sheath in barley leaves with changed export or photosynthetic rate. Plant Physiol. 118, 1525–1532. 10.1104/pp.118.4.1525 9847129 PMC34771

[B37] Koroleva O. A. Tomos A. D. Farrar J. F. Roberts P. Pollock C. J. (2000). Tissue distribution of primary metabolism between epidermal, mesophyll and parenchymatous bundle sheath cells in barley leaves. Aust. J. Plant Physiol. 27, 747–755. 10.1071/PP99156

[B38] Leidreiter K. Kruse A. Heineke D. Robinson D. G. Heldt H. W. (1995). Subcellular volumes and metabolite concentrations in potato (*Solanum tuberosum* cv. Désirée) leaves. Bot. Acta 108, 439–444. 10.1111/j.1438-8677.1995.tb00518.x

[B39] Liu D. Y. T. Smith P. M. C. Barton D. A. Day D. A. Overall R. L. (2015). Characterisation of Arabidopsis calnexin 1 and calnexin 2 in the endoplasmic reticulum and at plasmodesmata. Protoplasma 254, 125–136. 10.1007/s00709-015-0921-3 26680228

[B40] Liu L. Chen X. (2018).Intercellular and systemic trafficking of RNAsin plants. Nat. Plants 4, 869–878. 10.1038/s41477-018-0288-5 30390090 PMC7155933

[B41] Maksimova A. I. Berke L. Salgado M. G. Klimova E. A. Romanova M. A. Pawlowski K.. (2021). What can the phylogeny of class I KNOX genes and their expression patterns in land plants tell us about the evolution of shoot development? Bot. J. Linn. Soc. 195, 254–280. 10.1093/botlinnean/boaa088

[B42] McCauley M. M. Evert R. F. (1989). Minor veins of the potato (*Solanum tuberosum* L.) leaf: ultrastructure and plasmodesmatal frequency. Bot. Gazette 150:351–368. 10.1086/337781

[B43] Modla S. Caplan J. L. Czymmek K. J. Lee J.-Y. (2020). Localization of fluorescently tagged protein to plasmodesmata by Correlative Light and Electron Microscopy, in Plasmodesmata: Methods and Protocols, Methods in Molecular Biology, vol. 1217, eds Heinlein M., 121–133. 25287200 10.1007/978-1-4939-1523-1_8

[B44] Omidbakhshfard M. A. Sokolowska E. M. Di Vittori V. de Souza L.P. Kuhalskaya A. Brotman Y.. (2020). Multi-omics analysis of earlyleaf development in *Arabidopsis thaliana*. Patterns 2:100235. 10.1016/j.patter.2021.100235 33982025 PMC8085607

[B45] Oparka K. J. Roberts A. G. Boevink P. Santa Cruz S. Roberts I. Pradel K. S.. (1999). Simple, but not branched, plasmodesmata allow the non-specific trafficking of protein in developing tobacco leaves. Cell 97, 743–754. 10.1016/S0092-8674(00)80786-2 10380926

[B46] Poethig W. S. (1987). Clonal analysis of cell lineage patterns in plant development. Am. J. Bot. 74, 581–594. 10.1002/j.1537-2197.1987.tb08679.x

[B47] R Core Team (2020). R: A Language and Environment for Statistical Computing. Vienna: R Foundation for Statistical Computing. Available online at: https://www.R-project.org/

[B48] Radford J. E. White R. G. (1998). Localization of a myosin-like protein to plasmodesmata. Plant J. 14, 743–750. 10.1046/j.1365-313x.1998.00162.x 9681037

[B49] Reichelt S. Knight A. E. Hodge T. P. Baluska F. Samaj J. Volkmann D.. (1999). Characterization of the unconventional myosin VIII in plant cells and its localization at the post-cytokinetic cell wall. Plant J. 19, 555–567. 10.1046/j.1365-313X.1999.00553.x 10504577

[B50] Riesmeier J. W. Willmitzer L. Frommer W. B. (1994). Evidence for an essential role of the sucrose transporter in phloem loading and assimilate partitioning. EMBO J. 13, 1–7. 10.1002/j.1460-2075.1994.tb06229.x 8306952 PMC394773

[B51] Robards A. W. (1976). Plasmodesmata in higher plants, in Intercellular Communication in Plants: Studies on Plasmodesmata, eds Gunning B. E. S. Robards A. W. (Berlin; Heidelberg: Springer). 10.1007/978-3-642-66294-2_2

[B52] Romanova M. A. Maksimova A. I. Pawlowski K. Voitsekhovskaja O. V. (2021). YABBY genes in the development and evolution of land plants. Int. J. Mol. Sci. 22:4139. 10.3390/ijms22084139 33923657 PMC8074164

[B53] Satina S. Blakeslee A. F. (1941). Periclinal chimeras in *Datura stramonium* in relation to development of leaf and flower. Amer. Jour. Bot. 28, 862–871. 10.1002/j.1537-2197.1941.tb11017.x

[B54] Schubert M. Koteyeva N. K. Zdyb A. Santos P. Voitsekhovskaja O. V. Demchenko K. N.. (2013). Lignification of cell walls of infected cells in *Casuarina glauca* nodules that depend on symplastic sugar supply is accompanied by reduction of plasmodesmata number and narrowing of plasmodesmata. Physiol. Plant. 147, 524–540. 10.1111/j.1399-3054.2012.01685.x 22924772

[B55] Sharman B. C. (1945). Leaf and bud initiation in the Gramineae. Bot. Gaz. 106, 269–289. 10.1086/335298

[B56] Stewart R. N. Burk L. G. (1970). Independence of tissues derived from apical layers in ontogeny of the tobacco leaf and ovary. Am. J. Bot. 57, 1010–1016. 10.1002/j.1537-2197.1970.tb09902.x

[B57] Stumpe M. Göbel C. Demchenko K. Hoffmann M. Klösgen R. B. Pawlowski K.. (2006). Identification of an allene oxide synthase (CYP74C) that leads to formation of α-ketols from 9-hydroperoxides of linoleic and linolenic acid in below-ground organs of potato. Plant J. 47, 883–896. 10.1111/j.1365-313X.2006.02843.x 16899083

[B58] Sud R. M. Dengler N. G. (2000). Cell lineage of vein formation in variegated leaves of the C4 grass *Stenotaphrum secundatum*. Ann. Bot. 86, 99–112. 10.1006/anbo.2000.1165

[B59] Tomos A. D. Hinde P. Richardson P. Pritchard J. Fricke W. (1994). Microsampling and measurement of solutes in single cells, in Plant Cell Biology—A Practical Approach, eds Harris N. Oparka K. J. (Oxford: IRL Press), 297–314.

[B60] Trivett C. L. Evert R. F. (1998). Ontogeny of the vascular bundles and contiguous tissues in the barley leaf blade. Int. J. Plant Sci. 159, 716–723. 10.1086/297589

[B61] Turgeon R. Medville R. (2004). Phloem loading: a revaluation of the relationship between plasmodesmatal frequencies and loading strategies. Plant Physiol. 136, 3795–3803. 10.1104/pp.104.042036 15516516 PMC527176

[B62] Van Bel A. J. E. Gamalei V. Y. (1992). Ecophysiology of phloem loading in source leaves. Plant Cell Environ. 15, 265–270. 10.1111/j.1365-3040.1992.tb00973.x

[B63] Voitsekhovskaja O. V. Koroleva O. A. Batashev D. R. Knop C. Tomos A. D. Gamalei Y. V.. (2006). Phloem loading in two Scrophulariaceae species. What can drive symplastic flow via plasmodesmata? Plant Physiol. 140, 383–395. 10.1104/pp.105.068312 16377750 PMC1326059

[B64] Voitsekhovskaja O. V. Rudashevskaya E. L. Demchenko K. N. Pakhomova M. V. Batashev D. R. Gamalei Y. V.. (2009). Evidence for functional heterogeneity of sieve element-companion cell complexes in minor vein phloem of *Alonsoameridionalis*. J. Exp. Bot. 60, 1873–1883. 10.1093/jxb/erp074 19321649

[B65] Wickham H. (2016). ggplot2: Elegant Graphics for Data Analysis. New York, NY: Springer. 10.1007/978-3-319-24277-4_9

[B66] Wickham H. Francois R. Henry L. Muller K. (2020). dplyr: A Grammar of Data Manipulation. R package version. 1.0.2. Available online at: https://CRAN.R-project.org/package=dplyr

[B67] Wickham H. Bryan J. (2019). readxl: Read Excel Files. R package version 1.3.1. Available online at: https://CRAN.R-project.org/package=readxl (accessed March 29, 2021).

[B68] Winter H. Robinson D. G. Heldt H. W. (1993). Subcellular volumes and metabolite concentrations in barley leaves. Planta 191, 180–190. 10.1007/BF00199748

[B69] Winter H. Robinson D. G. Heldt H. W. (1994). Subcellular concentrations and metabolite concentrations in spinach leaves. Planta 193, 530–535. 10.1007/BF0241155816668375

[B70] Xie Y. (2020). knitr: A General-Purpose Package for Dynamic Report Generation in R. R package version 1.30.

