# Supplementary figures and images for: Leaf Epidermis: The Ambiguous Symplastic Domain
Source: Front Plant Sci. 2021 Jul 29;12:695415. doi: 10.3389/fpls.2021.695415 (PMC8358407; doi:10.3389/fpls.2021.695415)

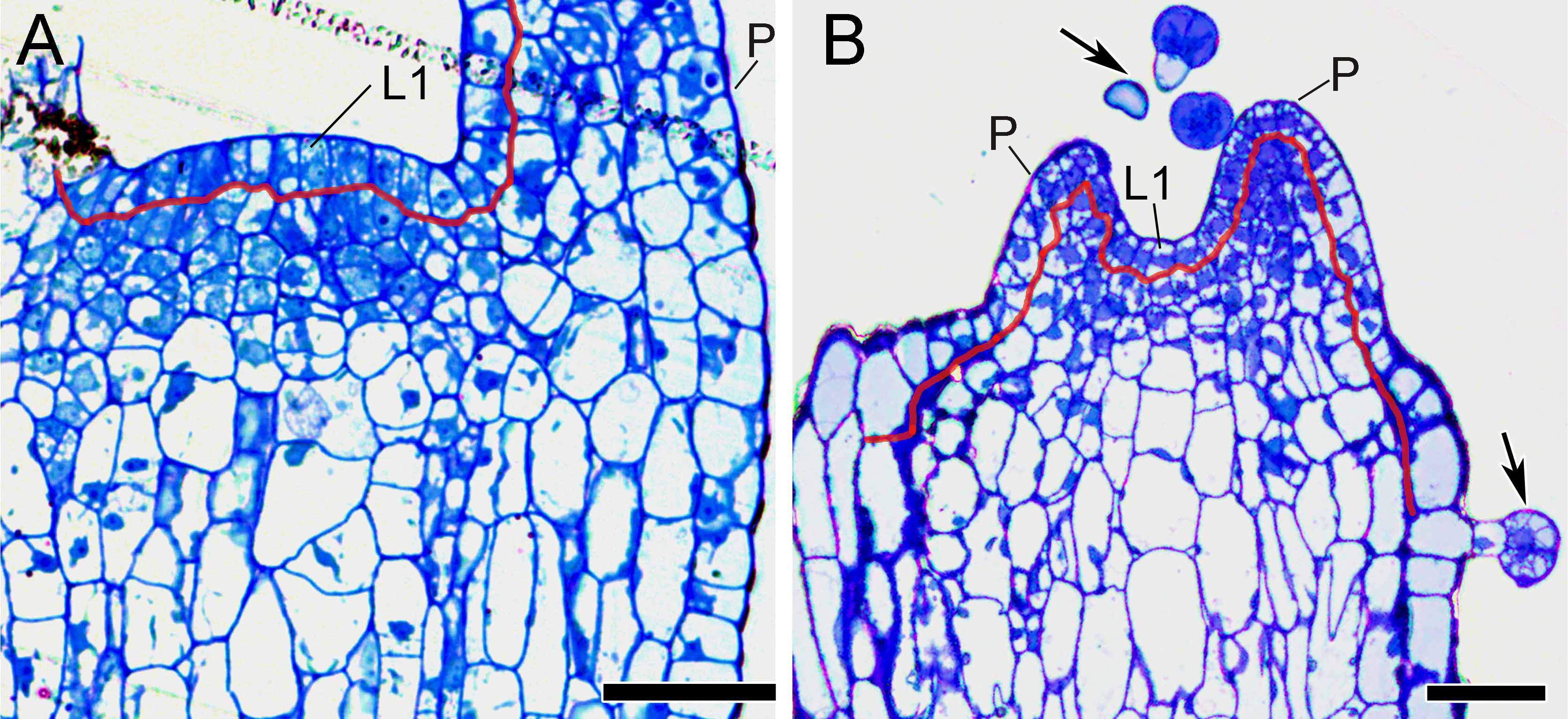

Supplement: Supplementary Figure 1 — Sections of shoot apices of Asarina barclaiana (A) and Alonsoa meridionalis (B). Within the shoot apical meristems, the tunica consists of the L1 layer which is continuous with the protoderm layer of leaf primordia (P) as outlined in red. Arrows point on glandular hairs. Shoot apices were embedded in epon resin and semi-thin sections were produced as described in Materials and Methods. The sections were stained with toluidine blue (0.05%). Scale bar: 20 μm. [file Image_1.JPEG]
